# Anti-Inflammatory Effect of Ebractenoid F, a Major Active Compound of *Euphorbia ebracteolata* Hayata, through Inhibition of Nuclear Factor-κB Activation

**DOI:** 10.3390/plants12152845

**Published:** 2023-08-01

**Authors:** Jaemoo Chun, Sang Yeon Mah, Yeong Shik Kim

**Affiliations:** 1 KM Convergence Research Division, Korea Institute of Oriental Medicine, Daejeon 34054, Republic of Korea; 2 Natural Products Research Institute, College of Pharmacy, Seoul National University, Seoul 08826, Republic of Korea

**Keywords:** ebractenoid F, *Euphorbia ebracteolata* Hayata, bioassay-guided fractionation, inflammation, NF-κB

## Abstract

*Euphorbia ebracteolata* Hayata (Euphorbiaceae family) is a perennial plant that is widely distributed in Korea, Japan, and China. Its roots contain bioactive diterpenes that have anti-inflammatory properties. However, the anti-inflammatory mechanisms are not yet fully understood. This study aimed to identify the most active anti-inflammatory compound from the roots of *E. ebracteolata* Hayata, using bioassay-guided fractionation and a combinative method of high-speed countercurrent chromatography (HSCCC) and preparative high-performance liquid chromatography (HPLC). Then, we investigated its anti-inflammatory mechanism in lipopolysaccharide (LPS)-stimulated RAW 264.7 macrophages. Ebractenoid F was identified as the most potent bioactive compound of *E. ebracteolata* Hayata. Ebractenoid F significantly decreased nitric oxide (NO) production and nuclear factor-κB (NF-κB) activation induced by LPS in RAW 264.7 macrophages. Moreover, ebractenoid F decreased the degradation of inhibitory κB-α, the nuclear translocation of the p65 and p50 subunits of NF-κB, and the expression of NF-κB downstream genes. Furthermore, ebractenoid F inhibited the phosphorylation of Akt and mitogen-activated protein kinases (MAPKs), such as extracellular signal-regulated kinase (ERK) and c-Jun NH_2_ terminal kinase (JNK), in LPS-stimulated RAW 264.7 cells. In conclusion, ebractenoid F exerts the most potent anti-inflammatory effect by suppressing NF-κB-mediated NO production in LPS-stimulated RAW 264.7 cells. Ebractenoid F may be a useful therapeutic compound for the prevention or treatment of inflammation-associated diseases.

## 1. Introduction

*Euphorbia ebracteolata* Hayata, which belongs to the Euphorbiaceae family, is widely distributed in China, Japan, and Korea. The roots of *E. ebracteolata* Hayata have been used for cutaneous tuberculosis, tumor, and chronic inflammation-associated diseases, such as tracheitis and psoriasis, in traditional Chinese medicine [1]. *E. ebracteolata* Hayata has been reported to contain bioactive substances, including diterpenes, flavonoids, and acetophenones [2]. Among the bioactive components, diterpenes from *E. ebracteolata* Hayata have been shown to have anti-inflammatory, antitumor, and antifungal activities [3,4]. However, the anti-inflammatory mechanisms of the bioactive components of *E. ebracteolata* Hayata are not yet fully understood.

Inflammation is a defensive process of the immune system that occurs in response to harmful stimuli. However, excessive inflammation causes significant tissue injuries and chronic inflammatory diseases, such as rheumatoid arthritis, asthma, and autoimmune diseases [5,6]. Nuclear factor-κB (NF-κB) is a transcription factor that plays a crucial role in inflammatory or immune responses through the regulation of proinflammatory factors, such as inducible nitric oxide synthase (iNOS) and cyclooxygenase-2 (COX-2) [7]. Under normal conditions, NF-κB forms a complex with the inhibitory κB (IκB)-α protein in the cytoplasm. However, in response to lipopolysaccharide (LPS), IκB-α is phosphorylated and subsequently degraded. Thereafter, free NF-κB is translocated to the nucleus and activates the transcription of proinflammatory target genes [8]. Therefore, the suppression of NF-κB activation is a potential strategy for the prevention or treatment of NF-κB-related inflammatory diseases.

Bioassay-guided fractionation is the most common procedure to isolate and characterize bioactive compounds from natural products based on their biological activity. This method involves extraction, fractionation, the bioassay screening of each fraction, and the identification of each isolated compound as well as the evaluation of its pharmacological mechanism [9]. In this study, we identified the most active anti-inflammatory diterpene derived from the roots of *E. ebracteolata* Hayata, using bioassay-guided fractionation and a combinative method of high-speed countercurrent chromatography (HSCCC) and preparative high-performance liquid chromatography (HPLC). We aimed to investigate its anti-inflammatory mechanism via suppression of the NF-κB pathway in LPS-stimulated RAW 264.7 macrophages as a model of inflammation.

## 2. Results

### 2.1. Bioactive Screening of E. ebracteolata Hayata for Anti-Inflammatory Activity

**Figure 1:**
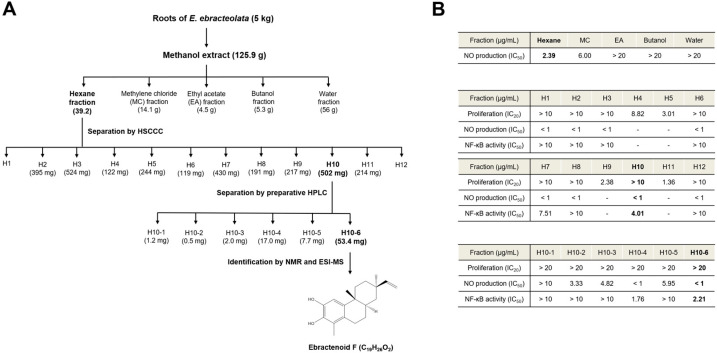
Outline of the bioassay-guided isolation of ebractenoid F from *Euphorbia ebracteolata* Hayata. (**A**) Extraction of fractions from *E. ebracteolata* Hayata and isolation of ebractenoid F. (**B**) IC_50_ values of the obtained fractions. Fractions were assessed in lipopolysaccharide (LPS)-stimulated RAW 264.7 macrophages. Cell proliferation, nitric oxide (NO) production, and nuclear factor-κB (NF-κB) activity were determined by the 3-(4,5-dimethylthiazol-2-yl)-2,5-diphenyltetrazolium bromide (MTT) assay, NO assay, and NF-κB secretory alkaline phosphatase (SEAP) assay, respectively. ESI-MS: electrospray ionization mass spectrometry; HPLC: high-performance liquid chromatography; HSCCC: high-speed countercurrent chromatography; NMR: nuclear magnetic resonance.

We conducted bioactive screening to identify the most active compound from *E. ebracteolata* Hayata. Initially, we evaluated the inhibitory effects of partitioned fractions from the methanol extract of *E. ebracteolata* Hayata on LPS-induced nitric oxide (NO) production in RAW 264.7 macrophages. Among the fractions, the hexane fraction showed the most potent inhibitory activity (IC_50_ = 2.39 μg/mL) on LPS-induced NO production. The most active hexane fraction was fractionated using HSCCC to obtain 12 subfractions. The subfractions were tested for the inhibition of NO production and NF-κB activity, using the NF-κB secretory alkaline phosphatase (SEAP) reporter gene assay in LPS-stimulated RAW 264.7 cells. At the same time, cell viability was assessed to exclude cytotoxic effects in RAW 264.7 cells. Most of the subfractions had an outstanding inhibitory effect on NO production, except the fractions that exhibited cytotoxicity. Importantly, the H10 subfraction had the most potent activity based on the inhibitory effect on NF-κB (IC_50_ = 4.01 μg/mL). To identify the active compounds, the H10 subfraction was further separated into six final fractions using preparative HPLC. Among them, H10-6 (ebractenoid F) was selected as the most potent component of *E. ebracteolata* Hayata, as it was the major component, showing high anti-inflammatory activity in terms of both NO production and NF-κB activity (Figure 1A,B).

### 2.2. Inhibitory Effect of Ebractenoid F on NO Production in LPS-Induced RAW 264.7 Cells

**Figure 2:**
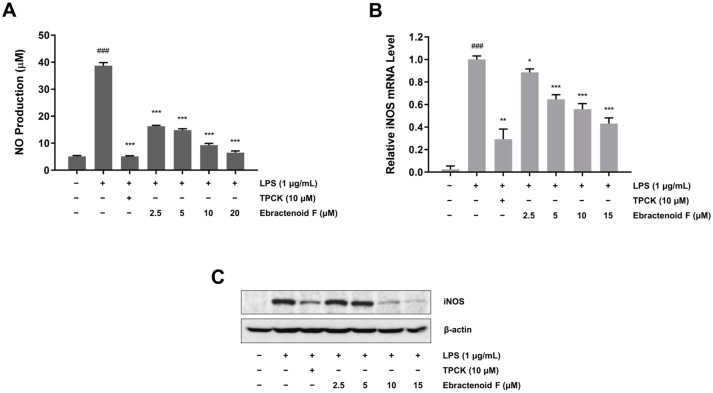
Inhibitory effect of ebractenoid F on NO production and inducible nitric oxide synthase (iNOS) expression in LPS-stimulated RAW 264.7 cells. (**A**) Cells were pretreated with ebractenoid F for 2 h and then stimulated with LPS for 18 h. The amount of NO in the medium was measured using the Griess reagent. (**B**) Cells were pretreated with ebractenoid F for 2 h and then stimulated with LPS for 12 h. Total RNA was isolated, and mRNA transcript levels were measured using quantitative reverse transcription polymerase chain reaction (qRT-PCR). For quantification, the mRNA expression data were normalized to β-actin. (**C**) Cells were pretreated with ebractenoid F for 2 h and then stimulated with LPS for 18 h. The expression of iNOS was determined by Western blotting. *N*-tosyl-L-phenylalanyl chloromethyl ketone (TPCK) was used as a positive control. The results are represented as the mean ± standard deviation (SD) from three independent experiments. ^###^ *p* < 0.001 indicates a significant difference compared with the unstimulated control group. * *p* < 0.05, ** *p* < 0.01, and *** *p* < 0.001 indicate significant differences compared with the LPS-treated group.

To explore the anti-inflammatory mechanism of ebractenoid F, we assessed the inhibitory effect of ebractenoid F on NO production. This effect was evaluated at noncytotoxic concentrations of ebractenoid F. RAW 264.7 cells were pretreated with various concentrations of ebractenoid F for 2 h and then treated with LPS (1 μg/mL) for 18 h. We found that LPS treatment dramatically increased NO production, while ebractenoid F significantly inhibited LPS-induced NO production in a dose-dependent manner (Figure 2A). Next, we investigated whether the inhibitory effect of ebractenoid F on NO production corresponded with the transcription level of iNOS in RAW 264.7 cells. The mRNA levels were assessed using quantitative reverse transcription polymerase chain reaction (qRT-PCR). The cells were pretreated with various concentrations of ebractenoid F for 2 h and then treated with LPS for 12 h. We found that ebractenoid F down-regulated the mRNA level of iNOS induced by LPS (Figure 2B). In addition, we investigated whether the inhibitory effect of ebractenoid F on NO production was related to a change in the expression of its synthesis enzyme iNOS. As shown in Figure 2C, LPS stimulation increased iNOS protein expression, which was undetectable in unstimulated cells. However, pretreatment with ebractenoid F dose-dependently suppressed LPS-induced iNOS protein expression. These results suggest that ebractenoid F exhibits anti-inflammatory activity by suppressing iNOS expression through transcriptional inhibition.

### 2.3. Inhibitory Effect of Ebractenoid F on LPS-Mediated NF-κB Transcriptional Activation in RAW 264.7 Cells

**Figure 3:**
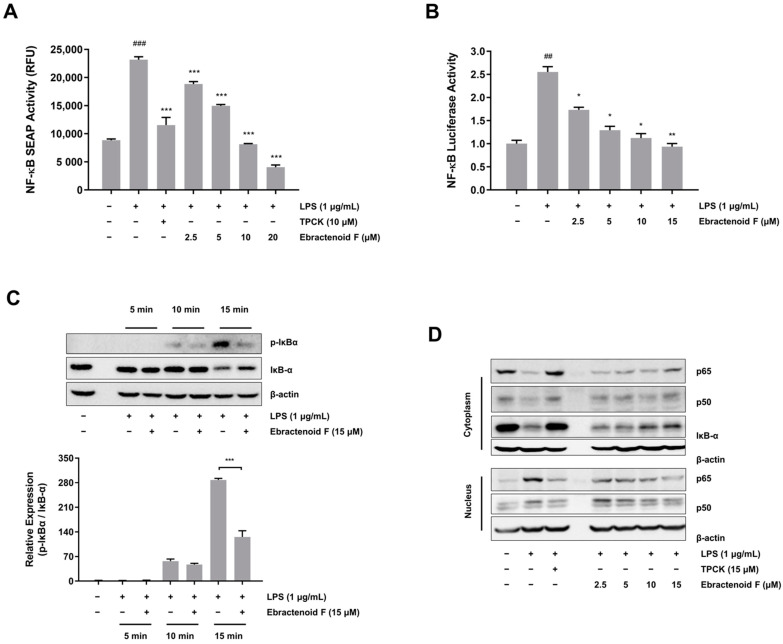
Inhibitory effect of ebractenoid F on NF-κB activation in LPS-stimulated RAW 264.7 cells. (**A**) Cells harboring the SEAP–neomycin phosphotransferase (NPT) reporter construct were pretreated with the indicated concentrations of ebractenoid F for 2 h and then exposed to LPS. The SEAP activity was measured in relative fluorescence units (RFU), using a microplate fluorometer. (**B**) Cells transfected with pCMV-Luc and pNF-κB-Luc reporter vector luciferase were pretreated with ebractenoid F for 2 h and then treated with LPS for 8 h. Luciferase activity was measured using a luminometer. (**C**) RAW 264.7 cells were pretreated with ebractenoid F for 2 h and then exposed to LPS for the specified time periods. The expressions of inhibitory κB (IκB)-α and p-IκB-α proteins in the cytoplasmic extracts were determined by Western blotting. The results were quantified using the ImageJ software. (**D**) Effect of ebractenoid F on NF-κB p65 localization to the nucleus. The cells were pretreated with ebractenoid F for 2 h prior to LPS treatment for 15 min. The cytoplasmic and nuclear extracts were prepared for Western blotting of p65 and p50. TPCK was used as a positive control. The results are represented as the mean ± SD from three independent experiments. ^##^ *p* < 0.01 and ^###^ *p* < 0.001 indicate significant differences compared with the unstimulated control group. * *p* < 0.05, ** *p* < 0.01, and *** *p* < 0.001 indicate significant differences compared with the LPS-treated group.

As NF-κB is the key regulator of iNOS, whether ebractenoid F affects NF-κB activity was investigated in LPS-stimulated RAW 264.7 cells. Initially, we examined NF-κB transcriptional activity using two different reporter gene assays (NF-κB SEAP and luciferase assays). We found that NF-κB-transfected RAW 264.7 cells had increased SEAP activity and luciferase activity stimulated by LPS (Figure 3A,B). However, pretreatment with ebractenoid F significantly suppressed NF-κB activity in LPS-stimulated RAW 264.7 cells. These results suggest that ebractenoid F suppresses iNOS expression through the attenuation of NF-κB transcription, which regulates the expression of proinflammatory genes. To better understand how ebractenoid F prevents LPS-induced NF-κB transcription, we examined the inhibitory effect of ebractenoid F on IκB-α degradation, which leads to the activation of NF-κB. RAW 264.7 cells were treated with ebractenoid F for 2 h and treated with LPS for 15 min. As shown in Figure 3C, ebractenoid F inhibited both the phosphorylation and degradation of IκB-α at 15 min after LPS treatment. In addition, the levels of p65 and p50 were decreased in the cytoplasm and increased in the nucleus after LPS treatment. However, pretreatment with ebractenoid F reversed these trends in a dose-dependent manner (Figure 3D and Figure S6), suggesting that ebractenoid F may inhibit NF-κB activation by blocking LPS-induced IκB-α degradation.

### 2.4. Inhibitory Effects of Ebractenoid F on the Inflammatory Mediators IL-6 and IL-1β

**Figure 4:**
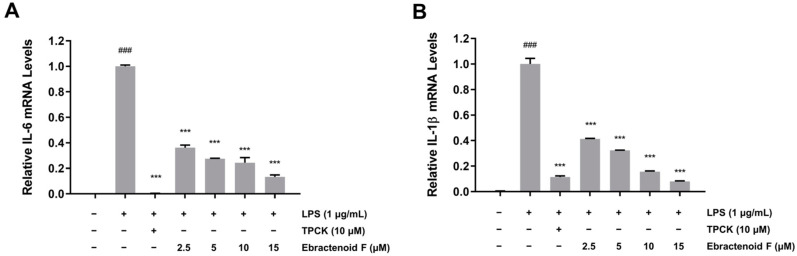
Inhibitory effects of ebractenoid F on the mRNA expressions of the proinflammatory mediators IL-6 (**A**) and IL-1β (**B**) in LPS-stimulated RAW 264.7 cells. Cells were pretreated with ebractenoid F for 2 h and then stimulated with LPS for 12 h. Total RNA was isolated, and the mRNA transcript levels were measured using qRT-PCR. For quantification, the mRNA expression data were normalized to β-actin. TPCK was used as a positive control. The results are represented as the mean ± SD from three independent experiments. ^###^ *p* < 0.001 indicates a significant difference compared with the unstimulated control group. *** *p* < 0.001 indicates a significant difference compared with the LPS-treated group.

Macrophages synthesize and release a variety of proinflammatory cytokines, such as IL-6 and IL-1β, which play crucial roles in the immune response. To determine the effects of ebractenoid F on the production of proinflammatory cytokines, mRNA expressions of IL-6 and IL-1β were determined using qRT-PCR. LPS alone significantly increased the mRNA expressions of IL-6 and IL-1β, but pretreatment with ebractenoid F dose-dependently suppressed the LPS-induced expressions of IL-6 and IL-1β (Figure 4A,B). These results suggest that ebractenoid F also suppresses the LPS-induced inflammatory response by inhibiting IL-6 and IL-1β.

### 2.5. Inhibitory Effect of Ebractenoid F on Phosphorylation in the Mitogen-Activated Protein Kinase (MAPK) and Akt Pathways

**Figure 5:**
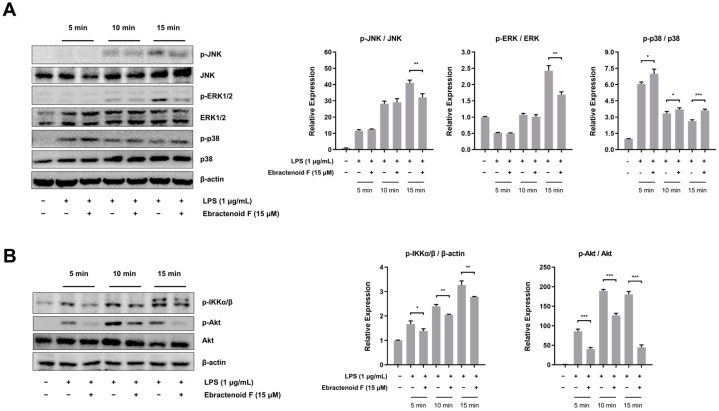
Inhibitory effect of ebractenoid F on the phosphorylation of mitogen-activated protein kinases (MAPKs) and Akt in LPS-stimulated RAW 264.7 cells. Cells were pretreated with ebractenoid F for 2 h and then stimulated with LPS for 5, 10, and 15 min. (**A**) The phosphorylation of MAPKs was determined by Western blotting with antibodies against c-Jun NH_2_ terminal kinase (JNK), p-JNK, extracellular signal-regulated kinase 1/2 (ERK1/2), p-ERK1/2, p38, and p-p38. (**B**) The phosphorylation of IκB kinase α/β (IKKα/β) and Akt was determined by Western blotting with antibodies against p-IKKα/β, p-Akt, and Akt. The results were quantified using the ImageJ software. * *p* < 0.05, ** *p* < 0.01, and *** *p* < 0.001 indicate significant differences compared with the LPS-treated group.

The MAPK pathway plays an important role in the release of proinflammatory cytokines and mediators by transcriptional regulation of NF-κB in LPS-stimulated RAW 264.7 cells [10]. To investigate the upstream signaling molecules related to NF-κB inactivation, we examined the effects of ebractenoid F on the phosphorylation of c-Jun NH_2_ terminal kinase (JNK), extracellular signal-regulated kinase 1/2 (ERK1/2), and p38 in the presence of LPS. We found that the phosphorylation of JNK, ERK1/2, and p38 was increased from 5 min to 15 min after LPS treatment. However, pretreatment with ebractenoid F down-regulated the phosphorylation of JNK and ERK1/2, but not p38 in LPS-stimulated RAW 264.7 cells (Figure 5A). Akt also has been shown to be associated with the activation of IκB kinase (IKK) and NF-κB [11]. To confirm the effect of ebractenoid F on the Akt pathway, we examined the phosphorylation of p-IKKα/β and Akt. As shown in Figure 5B, ebractenoid F decreased the phosphorylation of IKKα/β and Akt. These results suggest that ebractenoid F attenuates the function of the proinflammatory transcription factor NF-κB by inhibiting the ERK1/2, JNK, and Akt signaling pathways.

## 3. Discussion

The roots of *E. ebracteolata* Hayata are a rich source of diterpenes, which have diverse biological activities, such as anti-inflammatory, anticancer, and antimicrobial effects. Many diterpenes have been isolated from the roots of *E. ebracteolata*, including rosane-type [4], isopimarane-type [12], casbane-type [13], and abietane-type [2] diterpenes. Interestingly, many abietane-type diterpenes exhibit various pharmacological activities. For instance, tanshinone IIA, the main active diterpene from *Salvia miltiorrhiza*, is used in many therapeutic remedies in traditional Chinese medicine [14]. Carnosic acid, which is highly abundant in rosemary (*Rosmarinus officinalis*), possesses various pharmacological properties, including anti-inflammatory, anticancer, and anti-oxidative effects [15]. However, research on rosane-type diterpenes is relatively scarce. Ebractenoid F, which is a rosane-type diterpene, was found to exhibit effective anti-inflammatory properties and identified as an active constituent of *E. ebracteolata* Hayata from bioactivity-directed fractionation. Specifically, both H10-4 and H10-6 showed anti-inflammatory activities by inhibiting NO production and NF-κB activation, with H10-4 exhibiting even stronger anti-inflammatory activity. Nevertheless, the amount of H10-6 was approximately three times greater than that of H10-4. Therefore, H10-6 was selected for further investigation to explore its bioactive mechanism, and we identified ebractenoid F as a potential anti-inflammatory component. Ebractenoid F has been reported to decrease NO production in LPS-stimulated macrophages [4]. However, the mechanism underlying the anti-inflammatory effect of ebractenoid F on LPS-induced inflammation remains unknown. HSCCC has many advantages, such as high recovery of liquid–liquid extraction, ease of scale-up, and low solvent consumption [16,17]. For effective separation, a combined method involving HSCCC and preparative HPLC was applied in this study. This combination method proved highly effective in fractionating compounds from *E. ebracteolata* Hayata. As a result, ebractenoid F was successfully isolated using the established method. Our approach can be readily applied to rapidly separate ebractenoid F from *E. ebracteolata* Hayata and other diterpenes for further biological studies.

Macrophages play an important role in a host’s immune responses against bacterial infection through phagocytosis and intracellular killing [18]. Activated macrophages rapidly trigger the expression of a series of genes responsible for the release of proinflammatory mediators, including reactive oxygen and nitrogen species [19]. Among them, NO plays an important role in the regulation of inflammatory responses to infection. It has anti-inflammatory activity under normal physiological conditions. However, NO is regarded as a proinflammatory mediator that induces inflammation because of excessive production in abnormal physiological situations [20]. NF-κB is one of the transcription factors regulating iNOS and the subsequent production of NO in the immune response and under inflammatory conditions. When macrophages are activated by LPS, IκB-α is phosphorylated by IKK, ubiquitinated, and rapidly degraded, which leads to subsequent nuclear translocation of the p50 and p65 subunits of NF-κB [21]. Transcription factor NF-κB is considered one of the most important regulators of proinflammatory gene expression. Proinflammatory mediators, such as iNOS, IL-6, and IL-1β, are mediated by NF-κB [22]. A compound that was shown to interfere with NO or iNOS may act via the inhibition of NF-κB [23]. Thus, an NF-κB inhibitor can be useful for treating or preventing inflammatory diseases. In this study, we screened for bioactive components and identified ebractenoid F, which was found to suppress NF-κB activation and NO production in LPS-stimulated RAW 264.7 cells. Ebractenoid F suppressed NO production by inhibiting NF-κB activation. Moreover, ebractenoid F suppressed IκB-α degradation, nuclear translocation of NF-κB, and its promoter activity.

MAPKs comprise a family of highly conserved serine/threonine kinases that have been implicated as playing key regulatory roles in inflammatory signaling pathways induced by various stimuli [24]. JNK, ERK, and p38 are the main MAPKs involved in immune regulation and activation [25]. LPS was shown to activate JNK, ERK, and p38 through their phosphorylation to produce inflammatory mediators, such as iNOS, in a mouse macrophage cell line [26]. We found that ebractenoid F suppressed the phosphorylation of JNK and ERK1/2, but not p38, which was induced by LPS. Suppression of the phosphorylation of JNK and ERK1/2 by ebractenoid F may partially be responsible for its anti-inflammatory activity. However, anti-inflammatory substances might change gene expression through either the MAPK-dependent or -independent pathway [27]. More studies are needed to explore the mechanism of this potential association. Akt has been shown to phosphorylate IKK, which positively regulates the NF-κB signaling pathway [11]. Inhibition of Akt phosphorylation has been shown to decrease the phosphorylation of IκB and attenuate the degradation of IκB-α in LPS-stimulated macrophages [28]. We found that ebractenoid F suppressed the phosphorylation of IKKα/β and Akt in LPS-stimulated RAW 264.7 macrophages. Thus, ebractenoid F may modulate PI3K/Akt-regulated NF-κB activation. A limitation of this study is that the anti-inflammatory effect of ebractenoid F was investigated only on RAW 264.7 cells. It is necessary to perform further in vitro experiments and use in vivo models to validate the role of ebractenoid F in the observed anti-inflammatory properties.

## 4. Materials and Methods

### 4.1. Materials and Reagents

The dried roots of *E. ebracteolata* Hayata were purchased from Kyung Dong market (Seoul, Republic of Korea) and authenticated by Dr. Youngbae Suh, Seoul National University. A voucher specimen (NPRI-16-847) was deposited at the Herbarium of Natural Products Research Institute at Seoul National University. 3-(4,5-dimethylthiazol-2-yl)-2,5-diphenyltetrazolium bromide (MTT; #475989), LPS from *Escherichia coli* (#L2630), Griess reagent (#G4410), G418 (Geneticin; #G8168), and *N*-tosyl-L-phenylalanyl chloromethyl ketone (TPCK; #T4376) were purchased from Sigma-Aldrich (St. Louis, MO, USA). Dulbecco’s modified Eagle’s medium (DMEM; #CM002), penicillin–streptomycin (#CA005), and fetal bovine serum (FBS; #F0600) were purchased from GenDepot (Barker, TX, USA). All antibodies used were purchased from Santa Cruz Biotechnology (Santa Cruz, CA, USA).

### 4.2. Extraction and Isolation

The dried roots of *E. ebracteolata* Hayata (5 kg) underwent extraction with methanol by ultrasonication at room temperature for 12 h. The crude extract (125.9 g; 4.20% yield) was suspended in distilled water (DW) after evaporation under reduced pressure. Then, it was sequentially partitioned with hexane, methylene chloride, ethyl acetate, butanol, and DW. The hexane fraction (39.2 g) was dissolved in hexane:80% acetonitrile (1:1, *v*/*v*). The mobile (lower) phase was dried by a vacuum evaporator, suspended in ethanol, and then subjected to HSCCC for further isolation. HSCCC was filled with hexane as the stationary (upper) phase. The flow rate of the mobile phase was 3 mL/min, and the column rotation speed was 460 rpm. The HSCCC solvent system for the hexane fraction consisted of DW with 0.1% formic acid (eluent A) and acetonitrile with 0.1% formic acid (eluent B) with the following gradient conditions: 40–80% B at 0–200 min, 80–100% B at 200–250 min, and 100% B until 400 min. The subfractions were collected and analyzed by HPLC-UV. Twelve subfractions (H1–H12) were obtained using HSCCC from the hexane fraction. The H10 subfraction (502 mg) was dissolved in methanol, and six fractions (H10-1–H10-6) were obtained using preparative HPLC. The preparative HPLC solvent system for the H10 subfraction consisted of DW with 0.1% formic acid (eluent A) and acetonitrile with 0.1% formic acid (eluent B) with the following gradient conditions: 45–65% B at 0–15 min, 65–100% B at 15–40 min, and 100% B washing for 9 min. The final peak fractions (H10-1–H10-6) were collected according to the HPLC-UV chromatogram. HPLC-UV chromatogram of the H10-6 fraction is shown in Figure S1.

### 4.3. HPLC Analysis

Analyses were carried out by an INNO C_18_ column (5 μm; 4.6 mm × 150 mm). The injection volume was 20 μL. The mobile phase was optimized with DW with 0.1% formic acid (eluent A) and acetonitrile with 0.1% formic acid (eluent B) with the following gradient conditions: 45–65% B at 0–15 min, 65–100% B at 15–40 min, and equilibration with 100% B for 9 min at a flow rate of 0.8 mL/min. UV detection was conducted at 280 nm, and the column was maintained at room temperature.

### 4.4. Identification of the Isolated Compound

HPLC coupled with electrospray ionization mass spectrometry (ESI-MS) was used to characterize the isolated compound. The structure was elucidated by comparing the ^1^H and ^13^C nuclear magnetic resonance (NMR) spectra in CDCl_3_ with references. Two-dimensional NMR spectra were acquired on a Bruker Avance 500-MHz at the NCIRF (National Center for Interuniversity Research Facilities at Seoul National University). The structure of H10-6 was determined to be C_19_H_24_O_2_ by ESI-MS at *m*/*z* 287 [M + H]^+^ and NMR analysis (Figures S2–S4). The ^1^H and ^13^C NMR data showed that H10-6 is an 18-nor-rosane-type diterpenoid with an aromatic A-ring (Table 1). The relative configuration was determined by the ROESY spectrum (Figure S5). The correlations of Me-20/H-12*β*, H-12*β*/H-15, and H-8/Me-17 indicated a *β*-orientation for Me-20 and α-orientations for H-8 and Me-17. Thus, H10-6 was identified and elucidated as ebractenoid F (Figure 1A).

### 4.5. Cell Culture

RAW 264.7 cells derived from murine macrophages were purchased from the American Type Culture Collection (Manassas, VA, USA). Cells were maintained in DMEM supplemented with 10% FBS, 100 units/mL penicillin, and 100 μg/mL streptomycin at 37 °C in 5% CO_2_. RAW 264.7 cells harboring the pNF-κB SEAP–neomycin phosphotransferase (NPT) reporter construct were cultured under the same conditions, except that the medium was supplemented with 500 μg/mL of geneticin for these cells.

### 4.6. Cell Viability

Cell viability was measured using the MTT assay. RAW 264.7 cells were seeded into a 24-well plate at a density of 1 × 10^5^ cells/well and maintained for 24 h. Cells were pretreated with ebractenoid F for 2 h and treated with LPS (1 μg/mL) for another 18 h. The MTT solution (0.5 mg/mL) was added to each well, and the plate was incubated for 2 h. The formazan crystals were dissolved in dimethyl sulfoxide. The absorbance was measured at 595 nm using an Emax microplate reader (Molecular Devices, Sunnyvale, CA, USA). The relative cell viability was expressed as the percentage cell viability relative to the untreated control.

### 4.7. Measurement of NO Production

RAW 264.7 cells were seeded into a 24-well plate at a density of 1 × 10^5^ cells/well and maintained for 24 h. Cells were pretreated with the indicated concentration of ebractenoid F for 2 h and then treated with LPS (1 μg/mL) for 18 h. The culture medium was mixed with an equal volume of Griess reagent. The absorbance was measured at 540 nm using an Emax microplate reader. TPCK (10 μM) was used as a positive control. NO production in the culture medium was determined from a standard curve generated with sodium nitrite.

### 4.8. NF-κB SEAP Assay

The pNF-κB-SEAP-NPT plasmid activates the *SEAP* reporter gene in response to NF-κB activity and encodes the *NPT* gene for geneticin resistance. NF-κB transcriptional activity was measured using the *SEAP* reporter gene assay as previously described [29]. RAW 264.7 cells harboring the pNF-κB-SEAP-NPT plasmid were seeded into a 24-well plate at a density of 1 × 10^5^ cells/well and maintained for 24 h. Cells were pretreated with ebractenoid F for 2 h and then treated with LPS (1 μg/mL) for 16 h. The relative fluorescence units (RFU) from the product of the SEAP/MUP reaction were measured using Spectramax Gemini XS (Molecular Devices) at an excitation of 360 nm and an emission of 449 nm. TPCK (10 μM) was used as a positive control.

### 4.9. NF-κB Luciferase Assay

Plasmid DNA was purified using the DNA-spin^TM^ Plasmid DNA Purification Kit (iNtRON, Seongnam, Republic of Korea). Briefly, RAW 264.7 cells were transiently transfected with the pCMV-Luc and pNF-κB-Luc reporter vector using iN-fect™ in vitro Transfection Reagent (iNtRON). After incubation for 24 h, cells were pretreated with ebractenoid F for 2 h and then treated with LPS (1 μg/mL) for 8 h in 24-well plates. Cells were then lysed using passive lysis buffer (Promega, Madison, WI, USA). Luciferase activity was determined using a Dual-Luciferase Reporter Assay System (Promega) and measured with a luminometer (MicroLumat Plus, Berthold Technologies, Dortmund, Germany).

### 4.10. Western Blotting

For the cytoplasmic extract, cells were suspended in lysis buffer containing 10 mM HEPES (pH 7.9), 10 mM KCl, 0.2 mM ethylenediaminetetraacetic acid (EDTA), 0.1 mM ethylene glycol tetraacetic acid (EGTA), 1 mM dithiothreitol (DTT), and 1 mM phenylmethylsulfonyl fluoride (PMSF) on ice. After incubation for 15 min, 10% NP-40 was added, followed by centrifugation for 5 min. The supernatant was used as the cytoplasmic extract. The remaining pellets were resuspended in nuclear extraction buffer containing 20 mM HEPES (pH 7.9), 400 mM NaCl, 1 mM EDTA, 1 mM EGTA, 1 mM DTT, and 0.4 mM PMSF on ice for 1 h, followed by centrifugation for 10 min. The supernatant was used as the nuclear extract. For whole cell lysates, proteins were extracted using lysis buffer containing 20 mM HEPES (pH 7.6), 350 mM NaCl, 20% glycerol, 0.5 mM EDTA, 0.1 mM EGTA, 1% NP-40, 50 mM NaF, 0.1 mM DTT, and 0.1 mM PMSF. Proteins were quantified using the Bradford assay, loaded on 8% or 10% SDS polyacrylamide gel, and transferred to nitrocellulose membrane, which were blocked by 5% bovine serum albumin. The membranes were then incubated overnight at 4℃ with primary antibodies against iNOS (sc-7271), NF-κB p65 (sc-8008), NF-κB p50 (sc-8414), p-IκB-α (sc-8404), IκB-α (sc-1643), JNK (sc-7345), p-JNK (sc-6254), ERK1/2 (sc-514302), p-ERK1/2 (sc-7383), p38 (sc-7972), p-p38 (sc-7973), Akt (sc-5298), p-Akt (sc-514032), p-IKKα/β (sc-21661), and β-actin (sc-8432). Subsequently, the membranes were incubated with horseradish peroxidase-conjugated secondary antibodies for 1 h. The protein bands were visualized using a chemiluminescence kit (iNtRON).

### 4.11. qRT-PCR

Total RNA was extracted using Trizol reagent (Invitrogen, Carlsbad, CA, USA). The amount and purity of RNA were determined using a Nanodrop spectrophotometer (Thermo Fisher Scientific, Wilmington, DE, USA). A total of 1 μg of RNA was converted into cDNA with the amfiRivert Platinum cDNA Synthesis Master Mix (GenDepot). qRT-PCR was performed with a 7300 Real-Time PCR system (Applied Biosystems, Carlsbad, CA, USA) using SYBR Green (Bioneer, Daejeon, Republic of Korea). Relative gene expression was evaluated by the comparative CT method and normalized using β-actin. The following primer sequences were used for amplification: (1) iNOS: sense primer, 5′-TCC TAC ACC ACA CCA AAC-3′; antisense primer, 5′-CTC CAA TCT CTG CCT ATC C-3′; (2) β-actin: sense primer, 5′-CTG ACT ACC TCA TGA AGA TCC TC-3′; antisense primer, 5′-CAT TGC CAA TGG TGA TGA CCT G-3′; (3) IL-6: sense primer, 5′-AGG CTT AAT TAC ACA TGT TCT CTG G-3′; antisense primer, 5′-TTA TAT CCA GTT TGG TAG CAT CCA T-3′; (4) IL-1β: sense primer, 5′-GCC ACC TTT TGA CAG TGA TGA G-3′; antisense primer, 5′-AGT GAT ACT GCC TGC CTG AAG-3′.

### 4.12. Statistical Analysis

All the data are presented as the mean ± standard deviation (SD) from three independent experiments. A one-way analysis of variance followed by a Dunnett’s *t*-test was used to examine the significant differences between groups. A *p* value of <0.05 was considered statistically significant.

## 5. Conclusions

Ebractenoid F was identified as a major active compound of *E. ebracteolata* Hayata from bioactivity-directed fractionation. It suppresses NO production through the down-regulation of iNOS expression in LPS-stimulated RAW 264.7 cells. These effects of ebractenoid F are associated with the inhibition of NF-κB activation. Although further investigation is required to explore the upstream pathway of NF-κB in the anti-inflammatory effect of ebractenoid F, this study provides evidence for the possible use of *E. ebracteolata* Hayata and specifically ebractenoid F in the management of inflammatory-related diseases.

## Figures and Tables

**Table 1:** ^1^H and ^13^C nuclear magnetic resonance assignment of ebractenoid F (δ in ppm, 500 MHz for ^1^H, and 125 MHz for ^13^C).

Position	*δ*_H_ (*J* in Hz)	*δ*_C_
1	6.7, s	108.8
2	-	140.8
3	-	139.9
4	-	122.6
5	-	127.1
6	2.64, m	26.9
7	1.55, m	25.7
	1.64, m	
8	1.64, m	36.4
9	-	36.4
10	-	140.4
11	1.57, m	34.1
	1.95, m	
12	1.37, m	32.9
	1.66, m	
13	-	36.4
14	1.19, m	39.6
	1.44, m	
15	5.86, dd (17.4, 10.8)	151.1
16	4.87, dd (10.8, 0.9)	108.9
	4.95, dd (17.4, 0.9)	
17	1.02, s	22.8
18		
19	2.11, s	11.4
20	1.01, s	21.3

## Data Availability

The data presented in this research are available on request from the corresponding author.

## Supplementary Materials

The following supporting information can be downloaded at: https://www.mdpi.com/article/10.3390/plants12152845/s1, Figure S1: HPLC-UV chromatogram of the H10-6 fraction; Figure S2: LC-ESI/MS spectra and UV spectrum of ebractenoid F; Figure S3: ^1^H NMR spectra of ebractenoid F; Figure S4: ^13^C NMR spectra of ebractenoid F; Figure S5: ROESY spectrum of ebractenoid F; Figure S6: Quantification results of p65, p50, and IκB-α proteins in the cytoplasmic and nuclear extracts from RAW 264.7 macrophages.
