# Supplementary material for: Anti-Inflammatory Effect of Ebractenoid F, a Major Active Compound of Euphorbia ebracteolata Hayata, through Inhibition of Nuclear Factor-κB Activation
Source: Plants (Basel). 2023 Aug 1;12(15):2845. doi: 10.3390/plants12152845 (PMC10421244; doi:10.3390/plants12152845)
Supplement: Supplementary file 1 [file plants-12-02845-s001.zip › plants-2451235-supplementary.pdf]

plants

Supplementary Materials

Anti-Inflammatory Effect of Ebractenoid F, a Major Active Compound of *Euphorbia ebracteolata* Hayata, through Inhibition of Nuclear Factor-κB Activation

Jaemoo Chun [1,*], Sang Yeon Mah [2], Yeong Shik Kim [2,*]

[1] KM Convergence Research Division, Korea Institute of Oriental Medicine, Daejeon 34054, Republic of Korea
[2] Natural Products Research Institute, College of Pharmacy, Seoul National University, Seoul 08826, Republic of Korea
* Correspondence: jchun@kiom.re.kr (J.C.); kims@snu.ac.kr (Y.S.K.); Tel.: +82-42-868-9511 (J.C.); +82-2-880-2479 (Y.S.K.)

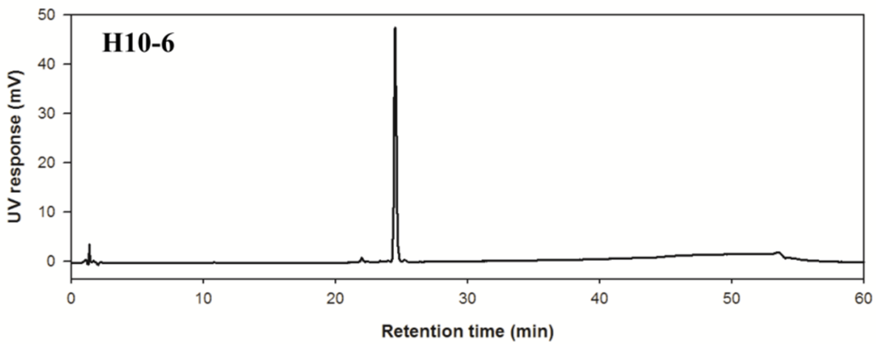

Figure S1. HPLC-UV chromatogram of H10-6 fraction.

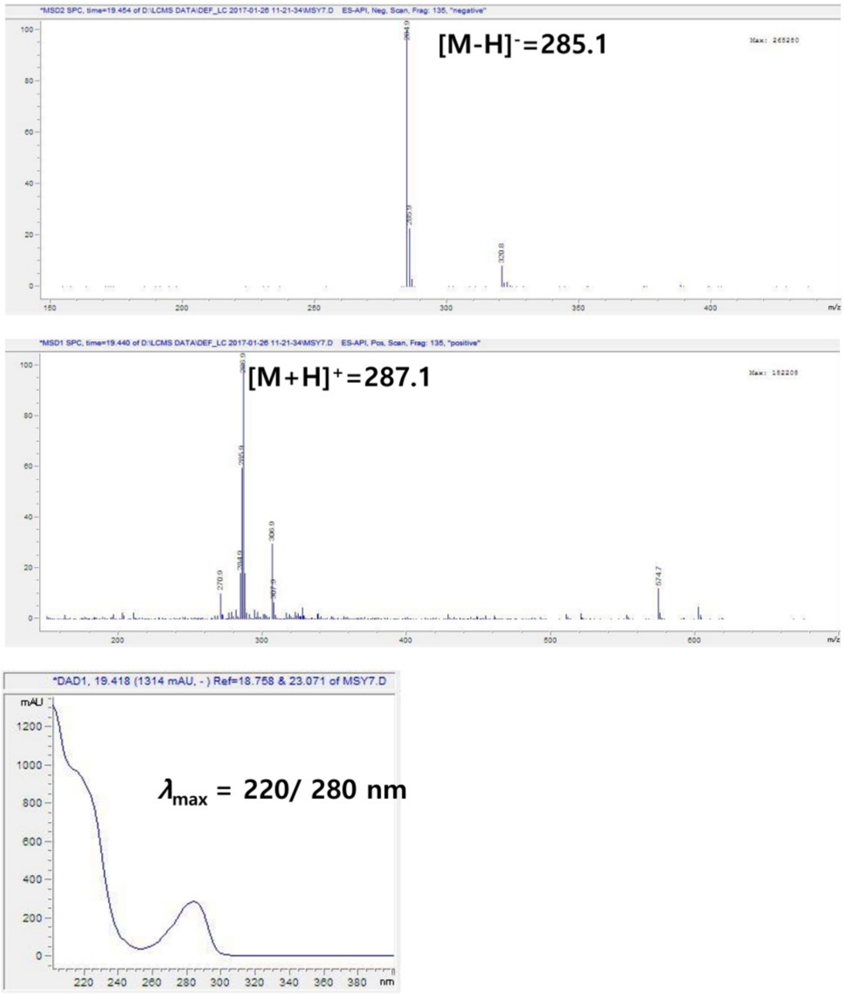

Figure S2. LC-ESI/MS spectra and UV spectrum of ebractenoid F. Ebractenoid F generated the protonated ions $[M+H]^+$ at *m/z* 287.1 in positive ion mode and the deprotonated ions $[M–H]^–$ at *m/z* 285.1 in negative ion mode. The compound has maximum UV absorption at 220 and 280 nm.

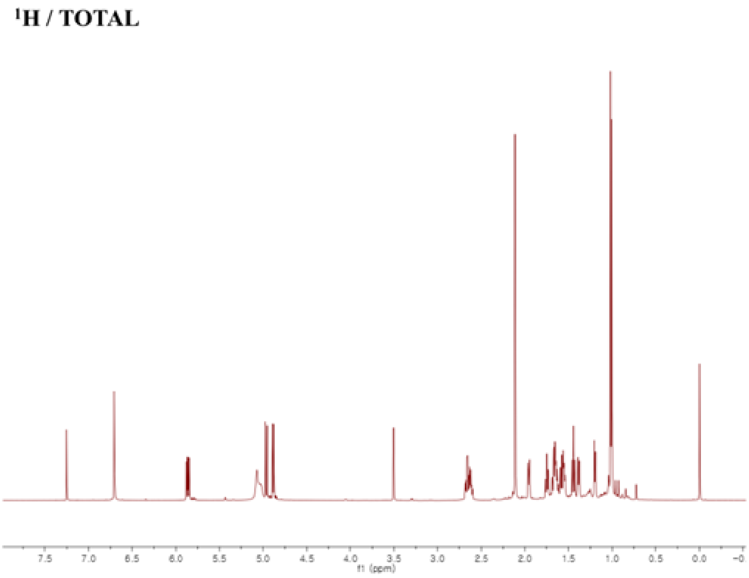

Figure S3. ^{1}H NMR spectra of ebractenoid F (Dissolved in $CDCl_3$, 500 MHz).

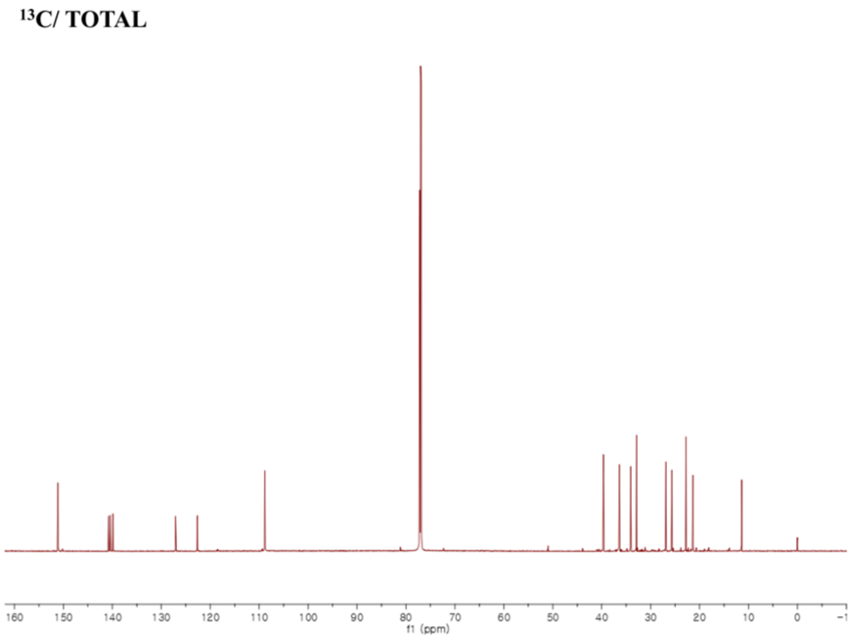

Figure S4. ^{13}C NMR spectra of ebractenoid F (Dissolved in $CDCl_3$, 125 MHz).

A/ ROESY

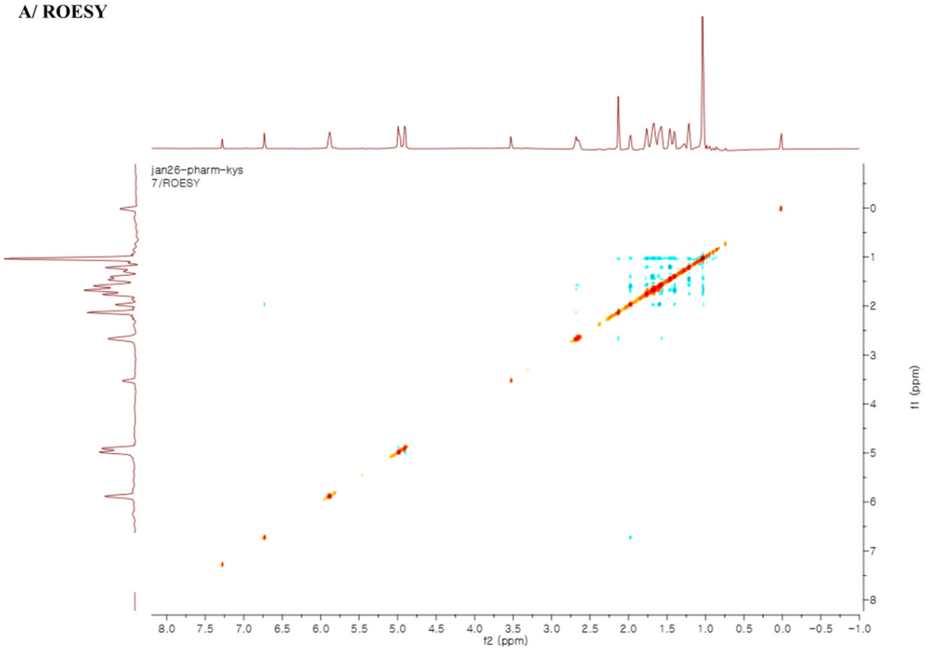

Figure S5. ROESY spectrum of ebractenoid F (Dissolved in $CDCl_3$, 500 MHz).

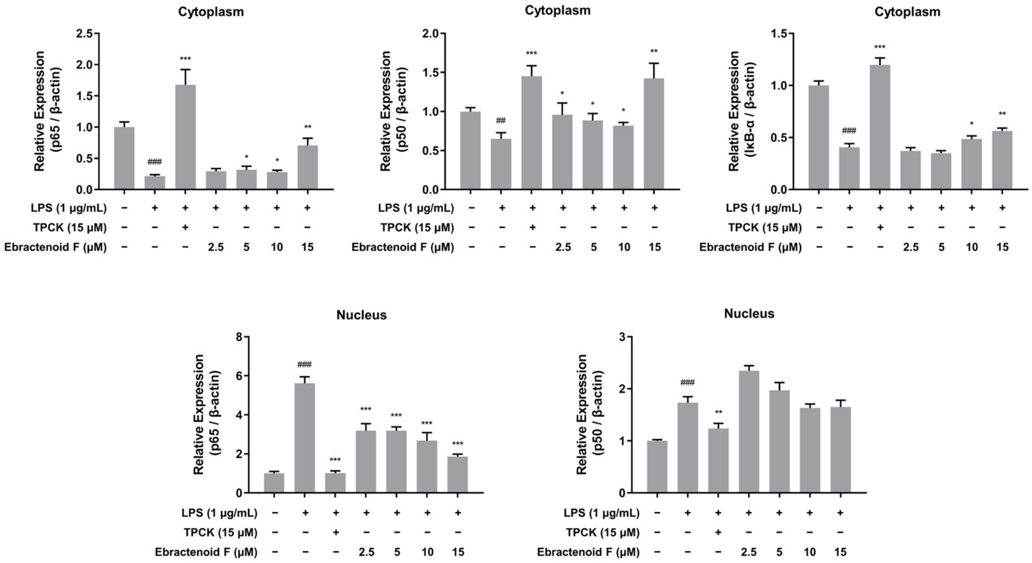

Figure S6. The quantification results of p65, p50, and IκB-α proteins in the cytoplasmic and nuclear extracts obtained from LPS-stimulated RAW 264.7 macrophages. The results were quantified using the ImageJ software.
